# Giant 2,150-gram bladder stone in a 32-year-old male: A case report from the Peruvian Amazon

**DOI:** 10.1016/j.radcr.2025.08.070

**Published:** 2025-09-24

**Authors:** Cintya Chavez-Fernandez, Laury Juarez-Barco, Sebastian Coronel-Arias, Elmer Paz-More, Jorge Fernández-Rosado, Raúl Hernán Sandoval-Ato

**Affiliations:** a Human Medicine Study Program, Universidad Privada Antenor Orrego, Avenida América Sur 3145, Urbanización Monserrate, Trujillo 13008, Perú; b Centro Médico Digital, Avenida Grau 831-841, Piura 20001, Perú; c Department of Internal Medicine, Hospital II-1 Moyobamba, Avenida Miguel Grau Cuadra 4, Moyobamba, San Martín 22201, Perú

**Keywords:** Urinary bladder calculi, Renal insufficiency, Hydronephrosis, Urinary tract infections

## Abstract

Giant bladder lithiasis is a rare clinical finding, particularly among young adults. We report the case of a 32-year-old male from the Peruvian Amazon who presented with progressive lower urinary tract symptoms, recurrent urinary tract infections, and weight loss over 2 years. Uroimaging revealed a solitary 15×15 cm hyperdense mass occupying the entire bladder cavity. Laboratory studies showed severe anemia, elevated creatinine, and urinary sediment with struvite crystals. Despite repeated evaluations, a timely diagnosis was delayed, leading to bilateral hydroureteronephrosis and renal impairment. The patient underwent open cystolithotomy, which extracted a 2150-gram stone in multiple fragments. Postoperative recovery was uneventful, and histopathology revealed bladder squamous metaplasia without malignancy. Follow-up imaging demonstrated residual bladder wall thickening and decreased renal dimensions. This case highlights the severe complications that can result from neglected bladder lithiasis in vulnerable populations and underscores the need for early urologic evaluation, especially in settings with limited access to healthcare.

## Introduction

Giant bladder lithiasis is an uncommon manifestation of urolithiasis, accounting for approximately 1% of cases, with stones over 500 grams reported only sporadically in the literature [1]. These stones are typically solitary and of mixed composition, often containing struvite, calcium oxalate, or ammonium phosphate crystals [2]. Their development is strongly associated with chronic urinary tract obstruction, neurogenic bladder, or recurrent urinary tract infections, particularly those involving urease-producing organisms such as Proteus mirabilis or Klebsiella pneumoniae [2,3]. Despite advances in diagnostic imaging and urologic care, giant bladder stones continue to be reported in resource-limited settings, where delays in access to health services remain a major barrier to early diagnosis and treatment. Clinically, patients with bladder lithiasis often present with nonspecific lower urinary tract symptoms including frequency, dysuria, suprapubic pain, and hematuria [4]. In extreme cases, complications such as bilateral hydronephrosis, obstructive uropathy, and chronic kidney disease may occur due to prolonged mechanical obstruction of the ureteral orifices. We report the case of a 32-year-old male from the Peruvian Amazon who developed a bladder stone measuring 15 × 15 cm and weighing 2,150 grams, 1 of the largest ever recorded in the region. The patient presented with a prolonged history of recurrent urinary tract infections and lower urinary symptoms, ultimately leading to severe renal dysfunction.

## Case report

A 32-year-old male was admitted to the urology department with a 2-year history of recurrent urinary tract infections despite antibiotic therapy, moderate hypogastric pain, leukocyturia, microscopic hematuria, dysuria, urinary urgency, pollakiuria, and loss of body weight.

He is an inmate of the penal facility in Moyobamba (Peruvian Amazon), with a medical history indicating smoking, and no notable surgical, familial or genetic history.

**Fig. 1:**
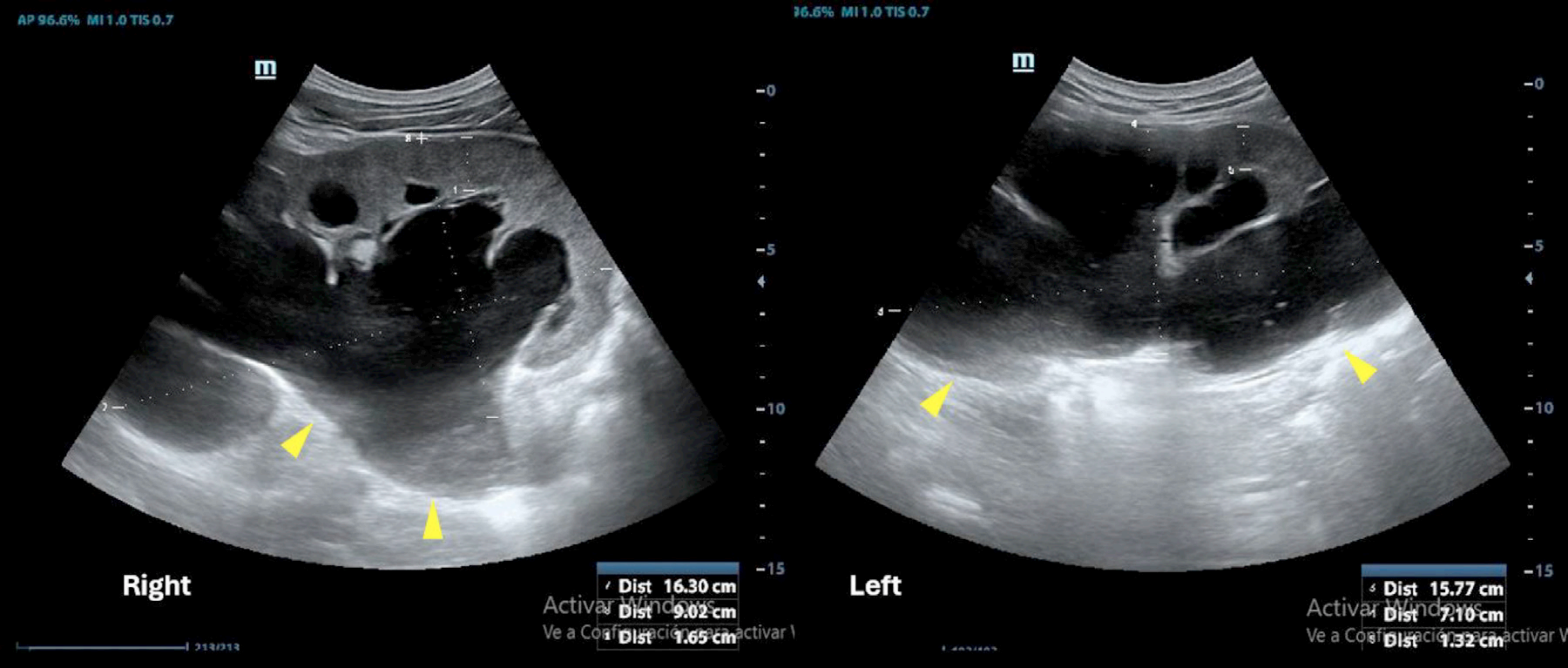
Renal ultrasound showed severe bilateral hydronephrosis.

During the physical examination, a hard suprapubic mass was noted, accompanied by moderate tenderness upon probing of the hypogastrium, flanks, and positive bilateral lumbar percussion. Diagnostic evaluations indicated lymphocytopenia (14%), a white blood cell count of 12.26×10³/µL, mild normochromic normocytic anemia (hemoglobin: 10.4 g/dL), serum creatinine level at 3.30 mg/dL, presence of ammonium-phosphate-magnesium crystals in the alkaline urinary sediment, and urine culture negative for pathogenic microorganisms. The bladder ultrasound was postponed due to the absence of distinct anatomical landmarks. The renal ultrasonography revealed significant bilateral hydronephrosis (Fig. 1).

**Fig. 2:**
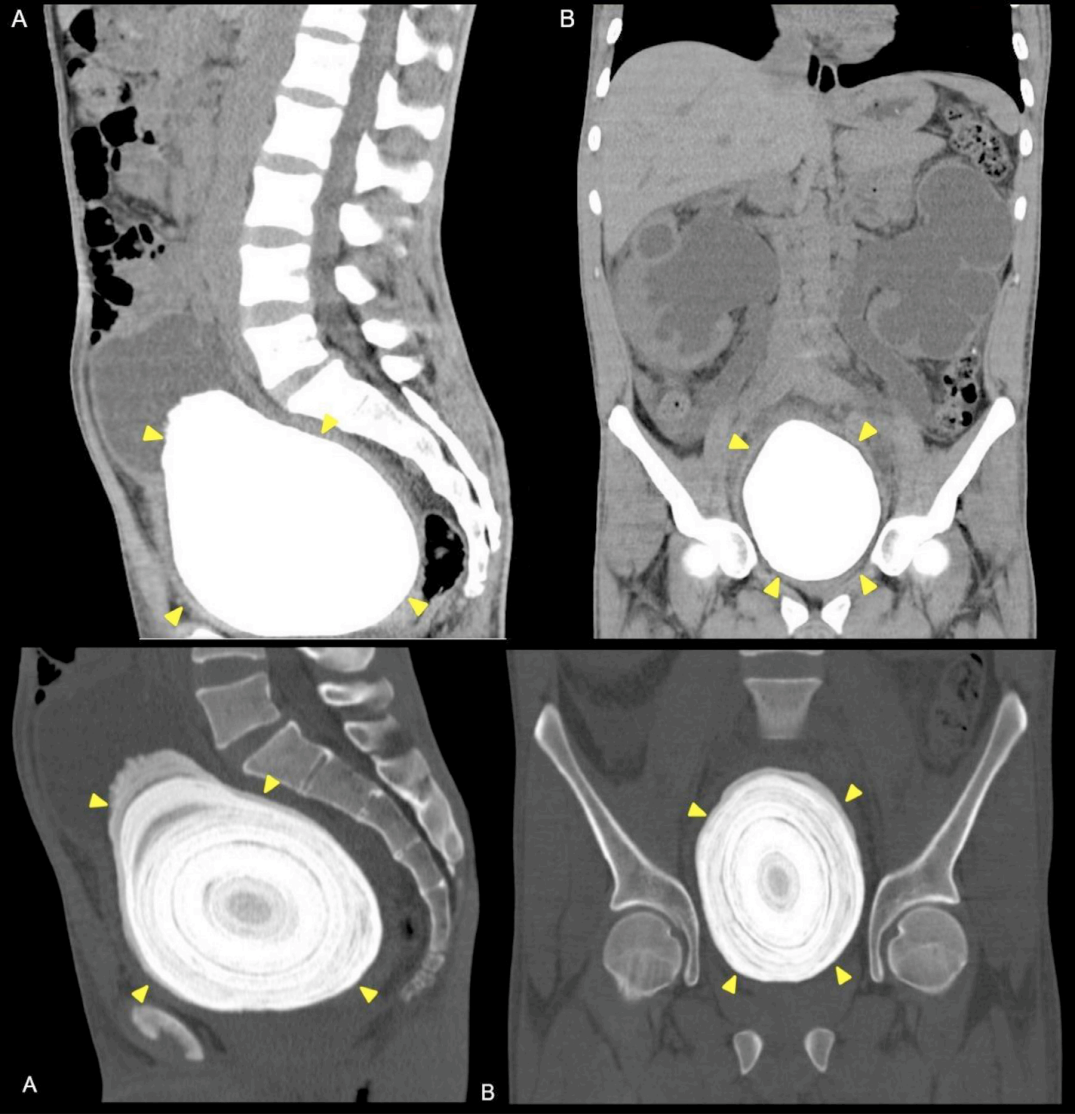
(A) Coronal, (B) Sagittal. A solid hyperdense mass with concentric layers is observed.

Nonenhanced computed tomography (NECT) demonstrated a solid hyperdense suprapubic mass exhibiting a calcified appearance, concentric layers, and regular edges encompassing the entire urinary bladder (Fig. 2); this mass obstructed the passage of the cystoscope through the bladder neck, confirming the diagnosis of bladder lithiasis.

**Fig. 3:**
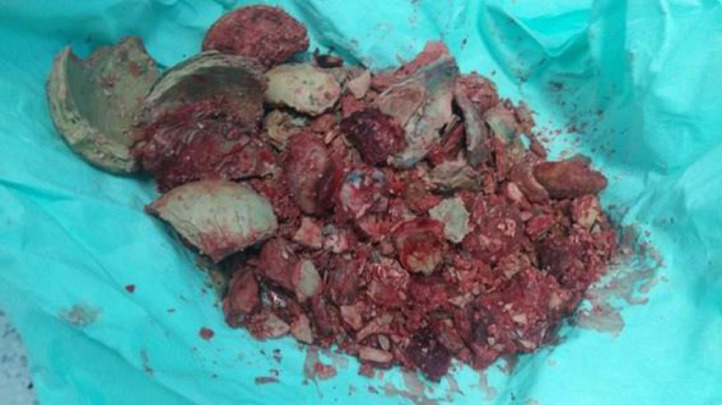
Postoperative finding: a fragmented giant bladder calculus is observed.

**Fig. 4:**
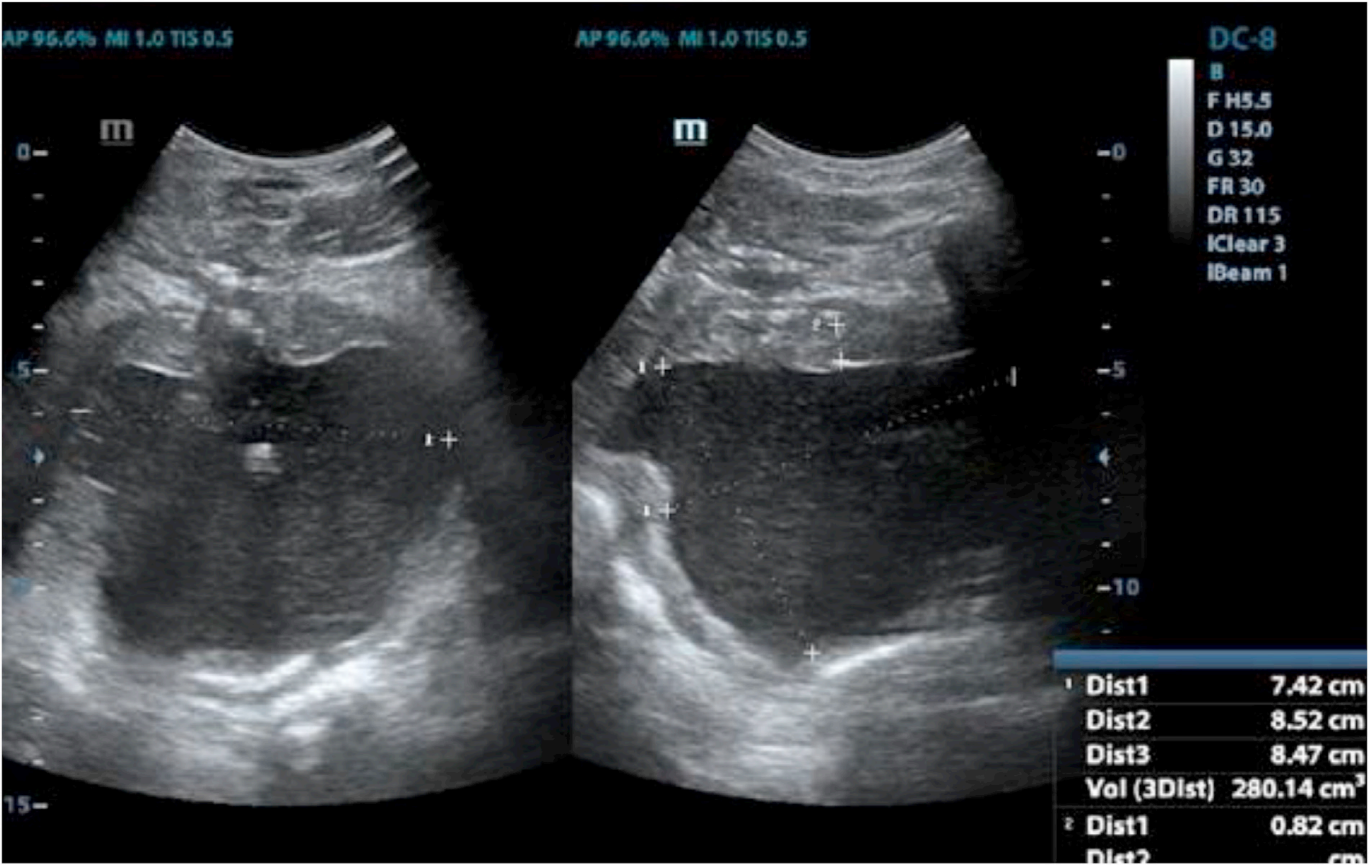
Postsurgical bladder ultrasound. Urinary bladder: Presents a diffuse wall thickening (up to 14 mm). Presence of 2 diverticula.

**Fig. 5:**
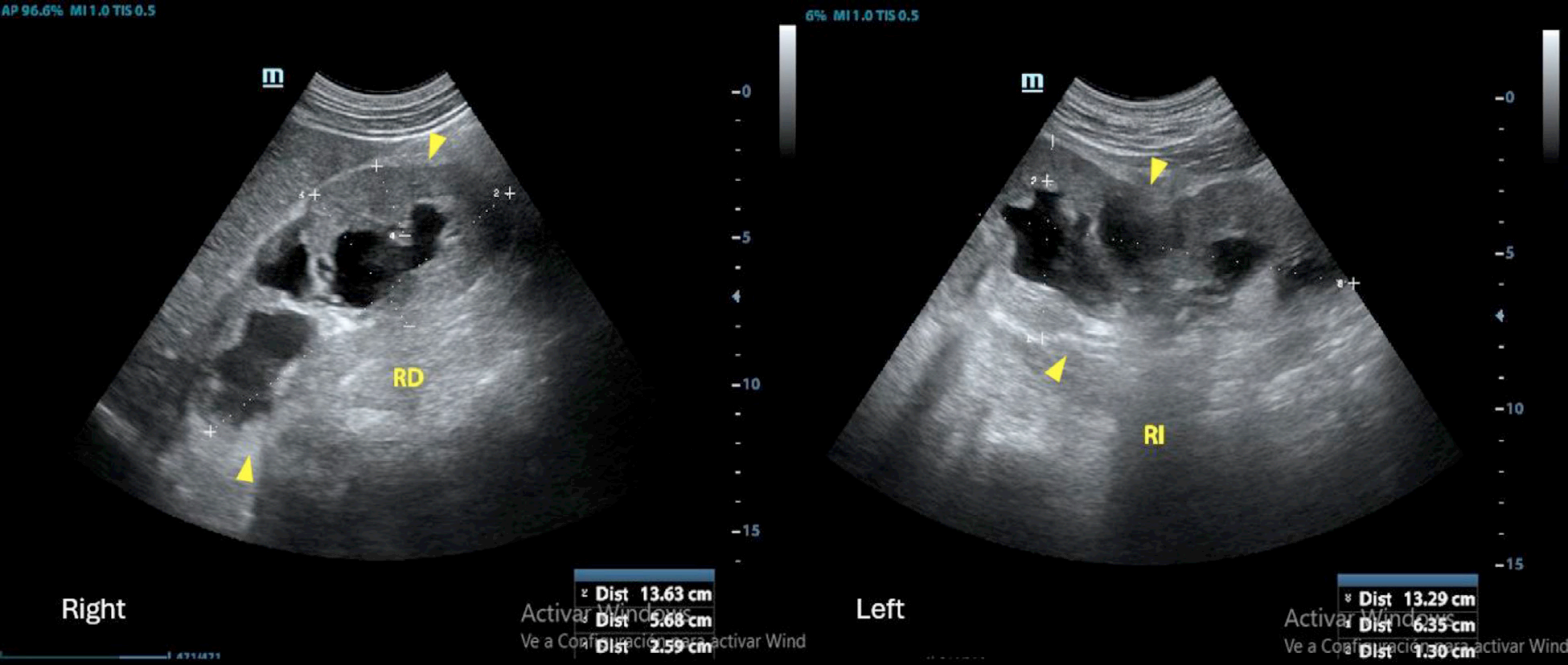
Postsurgical renal ultrasound. Mild and bilateral decrease in renal dimensions with signs of interstitial nephropathy .

A cystolithotomy was conducted via a median infraumbilical incision. A 15×15 cm bladder stone, firmly attached to its wall, was retrieved through fragmentation (Fig. 3), weighing 2150 grams. During the follow-up, the patient exhibited no complications postsurgery. Bladder squamous metaplasia, resulting from a large calculus and devoid of malignancy, was documented in the biopsy sample submitted. Serial urine cultures were conducted, yielding negative results for bacterial proliferation. On the twelfth day postsurgery, the patient was discharged from the hospital. Renal and bladder ultrasound revealed a slight bilateral reduction in renal size, accompanied by indications of interstitial nephropathy and structural alterations characterized by diffuse wall thickening (Figs. 4 and 5).

Twenty-two days postsurgery, the patient returned for follow-up care, exhibiting no discomfort, afebrile, and with a Foley catheter containing yellowish urine in the collection bag. Hemoglobin, urea, and creatinine values were 10.6 g/dL, 2.09 mg/dL, and 59.9 mg/dL, respectively, as sequelae of the indicated pathology. The most recent follow-up documented in the institutional system pertained to a urological consultation conducted fifteen days subsequent to this visit, in 2024. The patient indicated an absence of soreness at the incision site. No additional appointments were recorded following this referral.

## Discussion

Calculi situated in the bladder account for 5 percent of all urolithiasis [5]. Nonetheless, the actual incidence and prevalence of individuals classified as having big bladder lithiasis, defined as weighing above 100 grams or measuring more than 4 centimeters, remain exceedingly low [6].

A literature review was conducted in MEDLINE, PUBMED, and SCOPUS from inception to December 2024, identifying few structured case reports involving stones over 900 grams in the native bladder. The attributes of these patients are delineated in Table 1. It is important to highlight that, as of the review date, no stone heavier than the 1 found in our locality has been documented.

**Table 1:** Review of published case reports of giant lithiasis greater than 900 grams in native bladder.

Study	Age (years)	Sex	Background	Symptoms / Physical exam	Urea / Creatinine	Imaging findings	Stone weight (grams)	Follow up
Aliyu et al. [10]	48	M	Long-term frequency, dysuria, and poor urinary stream	Suprapubic mass	NLV / NLV	Giant vesical calculus^b^	1600	N/A
Chengquan et al. [9]	54	M	Nine years of history of urinary frequency and urgency / Heavy smoker	Suprapubic area hard on palpation	NLV / NLV	Renal stone, hydronephrosis of both kidneys, and ureterectasia^b^ Giant stone within the bladder^a^	1048	Hydronephrosis disappeared 4 weeks after discharge. Normal urodynamic and urinalysis.
Hasan et al. [7]	57	F	R-UTI	Hard, rock-like mass in the lower abdomen	105 / 6	Giant vesical Stone and severe upstream hydroureteronephrosis^c^	1300	Kidney function improvement.
Shrestha et al. [6]	52	M	No relevant history	Lower abdominal pain, dysuria, and pollakiuria	4.17 mmol/L 59.7 µmol/L	Hydronephrosis^b^ Giant, regular bladder stone^a^	950	Normal renal function at 3 months of follow-up.
Rahka et al. [8]	20	M	R-UTI / History of passing stones / Heavy smoker	Suprapubic solid mass	N/A	Giant-opaque-intravesical mass^a^	940	No urinary symptoms were shown at 6-month follow-up
Mohd et al. [4]	30	M	Intermittent hematuria	Suprapubic mass	1392 mg/dL / 53.8 mg/dL	Large bladder calculi^a^ww Bilateral gross hydronephrosis^b^	1000	N/A

Results obtained from the bibliographic research carried out in MEDLINE, PUBMED, and SCOPUS until December 2024. M, male; F, female; N/A, not available; R-UTI, recurrent urinary tract infection; NLV, normal laboratory value.

a Abdominopelvic X-ray image.

b Abdominopelvic ultrasound image.

c Image by Computerized Axial Tomography of the abdomen and pelvis. In all clinical cases, open cystolithotomy was chosen as the definitive treatment.

The etiopathogenesis of bladder lithiasis in general is generally ascribed to obstructive factors affecting the urine outflow tract, infections, impaired bladder emptying, foreign bodies, bladder diverticula, and even migration of renal or ureteral calculi [3]. In this patient, we identify recurrent urinary infections as a potential reason, as this characteristic is associated with stones that beyond the recognized threshold [7].

In their report, Rakha Sulthan et al. [8] detail a fisherman patient who exhibited sun exposure, an irregular diet, inadequate hydration intake, and a bladder stone weighing 940 grams. The elevated temperatures in the town of Moyobamba and the inadequate conditions of a prison, lacking essential necessities, may have significantly contributed to the development of the described patient’s severe calculus. Moreover, it has been indicated that smoking in patients with stones above 1000 grams is likely a contributing risk factor [9].

The patient presented with hypogastric discomfort, dysuria, urgency, frequency, and hematuria. Patients with masses exceeding 900 grams exhibited analogous typical symptoms for extended durations [[8], [9], [10]]. This report emphasizes the lack of acute urine retention despite the presence of giant bladder lithiasis, which instead resulted in severe hydroureteronephrosis caused by closure of the ureteral orifices.

The diagnostic plan for these instances included x-rays and ultrasounds, which exhibited acceptable sensitivity and specificity [11]. In our experience, access to ultrasonography is restricted at the primary level of care [12]; furthermore, these methods are operator-dependent.

We underscore that despite the execution of a more precise imaging investigation, such as computerized axial tomography, the diagnosis and treatment were postponed, hence elevating the likelihood of serious consequences [13,14]. With prompt care, they can resolve [15]; however, in our instance, renal failure as the primary consequence was irreversible, akin to the findings reported by Hasan et al [7].

Although the biopsy in our patient indicated squamous metaplasia, the potential association between persistent inflammation of the bladder epithelium resulting from lithiasis and the emergence of neoplasms has been shown [13]. We advocate for ongoing surveillance of these patients to identify concerning initial signs that facilitate appropriate targeted intervention.

## Conclusion

Giant bladder lithiasis is a rare clinical condition, particularly in young adults, and can lead to considerable morbidity if not recognized and treated swiftly. This case exemplifies the potential severity of chronic, untreated urinary tract infections in at-risk populations with restricted access to healthcare, resulting in the development of a 2150-gram bladder stone and irreparable kidney impairment. Ongoing monitoring is essential, not only to assess renal function but also to detect probable neoplastic change resulting from chronic bladder irritation.

## Limitations and future scope

This report outlines certain limitations intrinsic to the portrayal of specific situations. The results cannot be extrapolated to a wider population, and a definitive causal relationship between bladder stones and related clinical outcomes cannot be confirmed. Moreover, the absence of long-term follow-up constrains our capacity to evaluate renal recovery and the possible danger of neoplastic transformation. This case offers significant evidence by recording 1 of the largest bladder stones recorded in the region, underscoring the necessity of early detection and prompt management in resource-constrained environments. Further research and multicenter case series are required to more accurately delineate the risk factors, natural history, and effective follow-up protocols for patients with big bladder stones.

## Funding

The author(s) received no financial support for the research, authorship, and/or publication of this article.

## CRediT authorship contribution statement

All authors contributed equally to the work. Their contributions included conceptualization, methodology design, validation, investigation, writing the original draft, review and editing. All authors have read and approved the final version of the manuscript.

## Declaration of generative AI and AI-assisted technologies in the writing process

During the preparation of this work, the author(s) used Grammarly in order to improve readability. After using this tool, the author(s) reviewed and edited the content as needed and take(s) full responsibility for the content of the publication.

## Patient consent

The authors declare that informed consent was obtained from the patient for the publication of this case report and any accompanying images.
